# Epigenetics, Darwin, and Lamarck

**DOI:** 10.1093/gbe/evv107

**Published:** 2015-05-29

**Authors:** David Penny

**Affiliations:** Institute of Fundamental Sciences, Massey University, Palmerston North, New Zealand

**Keywords:** Darwinism, epigenetics, evolution, genetics, Lamarck

## Abstract

It is not really helpful to consider modern environmental epigenetics as neo-Lamarckian; and there is no evidence that Lamarck considered the idea original to himself. We must all keep learning about inheritance, but attributing modern ideas to early researchers is not helpful, and can be misleading.

In general, I like papers that challenge some (apparently) accepted ideas. Perhaps this is because I was strongly influenced by Popperian ideas while still an undergraduate. Karl Popper responded to the apparent dilemma of the early 20th century that Isaac Newton’s Laws had been “believed” to be scientific truths for over 200 years, and then Albert Einstein had suggested with his theory of relativity that Newton’s Laws were only very good approximations—at least at lower velocities. Popper from the mid-1930s (e.g., Popper 1981), as a philosopher of science who took science seriously, suggested that

- humans had no special knowledge or ability to accept a theory as true for all time, but
- we should always test our theories and keep learning—and
- we should use the best-tested theory at any particular time.

For ourselves, we are heretics in that we do not “believe” that any prokaryote (akaryote) to eukaryote transition has been established—it depends very much on the nature of the outgroup, and there are good reasons to suggest the whole issue is unresolved (Penny et al. 2014). Yes, there is some good data, for example, on membrane properties (Sojo et al. 2014), but these can always be placed in a more general context. So it is excellent that early authors, such as Lamarck, are undergoing studies that are expanding our knowledge. The concept of epigenetics has a long history (Waddington 1942) and is certainly now mainstream—though not everyone agrees (Ptashne 2013). We are all continuing to learn, late last year a paper appeared implicating histone phosphorylation with transcription (Basnet et al. 2014). As such, I welcome good challenges to accepted “beliefs”—we all might learn something.

Thus, I should welcome this paper (Skinner 2015)—but still think that the paper is problematic—perhaps I see it as not addressing quite the right issues, or not the right questions? There has been a recent controversy in Nature over whether evolutionary biology needs to be rethought (Laland et al. 2014 argue for rethinking, vs. Wray et al. 2014 arguing for continued updating and expanding of our knowledge). In this controversy, we are very much in favor of always continuing to update our knowledge—humans have no special facility to believe only completely correct hypotheses. Knowledge should never be static, so I basically agree with Wray et al. (2014) about continued learning.

Perhaps the main point for epigenetics is that without the right genetics (coding sequences) there will be no epigenetics. This does need to be made clear—genetics and epigenetics are not alternatives (see later). So I am not yet convinced that “epigenetics” (as suggested in fig. 1 of Skinner 2015) is really more fundamental/basic that genomes and DNA sequences. There is now good evidence for several aspects of epigenetics in all the main subgroups of eukaryotes (Penny et al. 2014); though we do need some additional information to increase our confidence here. So maybe the figure 1 of Skinner just needs updating? There is no evidence whether genetics (sequences) or epigenetics came first—though that does not seem to inhibit strongly held beliefs.

So first, epigenetics depends on having the right genetics—the right enzymes, so in that sense it is part of genetics and inheritance. For example, without the ability to methylate cytosine, or to modify histones (in eukaryotes), epigenetics as we know it would not be possible. In a recent publication (Penny et al. 2014) we point this out, and suggest that epigenetics must at least be a general (ancestral) feature of eukaryotes. Epigenetics includes methylation (of both DNA and histones), acetylation, and phosphorylation. One of the main uncertainties in eukaryotes has been the Excavates as to whether they had epigenetics, but some aspects (especially acetylation) certainly appear to occur in the Excavate group, such as in *Giardia* (Sonda et al. 2010), and histone lysine acetylation plays an essential role in regulating the life cycle from cyst to trophozoite. Acetylation has also been characterized in *Plasmodium* (Trenholme et al. 2014) and *Entamoeba* (Ramakrishnan et al. 2004). DNA methylation is known in many diverse eukaryotes from mammals to nematodes to ciliates, where methylation and hydroxymethylation are involved in DNA elimination (summarized in Yi 2012). Devanapally et al. (2015) have now identified ds-RNA going into the germ-line, and there are interesting developments (Jiméneza et al. 2015) about circuits of interactions of proteins. Davis et al. (2013) have shown that bacterial cells also have epigenetics, but still we must have the right genetics in order to have epigenetics. So I certainly agree with Skinner (2015) that epigenetics must always be considered in inheritance, and so we are still learning about heredity (including epigenetics).

Second, and despite some popular thought, there is no real evidence that Lamarck himself considered that the inheritance at acquired characters was original to himself (Burkhardt 1977)—it appeared to be “general knowledge/assumptions” of the time. And there are other aspects of Lamarck’s understanding of evolution that we would not accept—including the separate nature of plants and animals is one, and the possibility of many separate origins of life is another, and the resistance to accept species extinction is still another. Yes, we should give more credence to the contribution of the late 18th and early 19th century French biologists, including Lamarck—they were a very active group. On a related matter, Charles Darwin also considered that there was inheritance of acquired characters (Burkhardt et al. 2014; pp. 329, 373–376)—it was a fairly common early idea. There is quite a lot of early information on Darwin’s contribution to genetics in the broad sense (Liu et al. 2009) and evolution in particular (Penny 2009). But none of this should get in the way of accepting the contribution of earlier biologists.

Third, there is no discussion of purpose (or deliberate action) in evolution, and this aspect does need to be included. Does the author think that there is purpose in the epigenetic events, it appears not to be stated? Another way of putting this question (perhaps an extreme way) is whether the macromolecules “know” that they are helping the survival of the organism. We have assumed that the molecules that carry out (environmental) epigenetics have no idea whether or not they are helping or hindering in a particular case (Penny 2014). It may well be that certain epigenetic changes are selected for under some environments, but that will have been selected for previously. Anyway, it should be made clear in the original whether environmental epigenetics is usually/necessarily advantageous to the organism. This aspect does need to be discussed, and it is basic to modern uniformitarianism/actualism.

Fourth, there does need to be some discussion on how “words” (and concepts) are used in science. To some scientists, each word has just one unique meaning, and such authors might like the definitions in table 1 of Skinner (2015). Though perhaps to the majority, words have several different meanings, and we must be clear in explaining our usage. There are literally thousands and thousands of concepts, and we have to distinguish (and be precise) what we intend. Under this view there are far too many concepts to learn all the words (Nowak 2000). This (more usual) view accepts that we continue to learn with time, and be clear when we use words/concepts. So perhaps we need to be more flexible here?

Finally to summarize, I would strongly prefer to see our concept of inheritance updated to formally include epigenetics, including environmental epigenetics. However, I see no real advantage in trying to attribute this very modern idea to Lamarck, especially as he did not see the idea as unique to himself (Burkhardt 1977). The history of ideas (see Cannon 1960) is complex on this issue and there are several points that need to be clarified—particularly including any choice (or not) of whether any epigenetic mutations are automatically advantageous? Initially the ‘uniformitarian’ position (before Charles Darwin) was mainly nonevolutionary (antievolutionary?) and there is in principle room to keep learning. It is certainly very good that Skinner (2015) has raised some important issues; however, I do not think that he addresses real answers to these basic questions. It is time that we saw additional studies of early evolutionists and to some extent this is provided for Lamarck by Burkhardt (1977). But there are other early evolutionists too—including Darwin’s own grandfather Erasmus Darwin (King-Hele 1999). There is an opportunity for studying again some of these early evolutionists. They are interesting intermediates between the traditional view of continuing spontaneous generation and the more recent (late 17th century) idea that species were separately created. I guess that I see epigenetics (including environmental epigenetics) as continuing to learn about inheritance, rather than anything fundamentally new and different?
